# eHealth Communication Intervention to Promote Human Papillomavirus Vaccination Among Middle-School Girls: Development and Usability Study

**DOI:** 10.2196/59087

**Published:** 2024-10-28

**Authors:** Youlim Kim, Hyeonkyeong Lee, Jeongok Park, Yong-Chan Kim, Dong Hee Kim, Young-Me Lee

**Affiliations:** 1 College of Nursing Kosin University Busan Republic of Korea; 2 College of Nursing, Mo-Im Kim Nursing Research Institute Yonsei University Seoul Republic of Korea; 3 Department of Communication Yonsei University Seoul Republic of Korea; 4 College of Nursing Sungshin University Seoul Republic of Korea; 5 School of Nursing DePaul University Chicago, IL United States

**Keywords:** cervical cancer, human papillomavirus, vaccines, health communication, chatbot, artificial intelligence, adolescent, mobile phone

## Abstract

**Background:**

As the age of initiating sexual intercourse has gradually decreased among South Korean adolescents, earlier vaccination of adolescents for human papillomavirus (HPV) is necessary before their exposure to HPV. Health communication includes “cues to action” that lead to preventive health behaviors, and recently, social networking services, which operate with fewer time and space constraints, have been used in various studies as a form of eHealth communication.

**Objective:**

This study aims to investigate the feasibility and usability of an eHealth communication intervention for HPV vaccination in middle-school girls aimed at the girls and their mothers.

**Methods:**

The eHealth communication intervention for HPV vaccination was developed using a 6-step intervention mapping process: needs assessments, setting program outcomes, selection of a theory-based method and practical strategies, development of the intervention, implementation plan, and testing the validity of the intervention.

**Results:**

A review of 10 studies identified effective health communication messages, delivery methods, and theories for HPV vaccination among adolescents. Barriers including low knowledge, perceived threat, and the inconvenience of taking 2 doses of the vaccine were identified through focus groups, suggesting a need for youth-friendly and easy-to-understand information for adolescents delivered via mobile phones. The expected outcomes and the performance objectives are specifically tailored to reflect the vaccination intention. Behavior change techniques were applied using trusted sources and a health belief model. Health messages delivered through a KakaoTalk chatbot improved awareness and self-efficacy. Quality control was ensured with the use of a log system. The experts’ chatbot usability average score was 80.13 (SD 8.15) and the average score of girls was 84.06 (SD 7.61).

**Conclusions:**

Future studies need to verify the effectiveness of health communication strategies in promoting HPV vaccination and the effectiveness of scientific intervention using a chatbot as a delivery method for the intervention.

## Introduction

### Background

In South Korea, cervical cancer is a principal health concern, affecting 1 in 8 women diagnosed with cancer in their 20s or 30s [1]. The United States Food and Drug Administration recommends the human papillomavirus (HPV) vaccination before the first sexual contact to increase its effectiveness in preventing cervical cancer [2].

According to a study of HPV infection among adult women in South Korea [3], approximately 20,000 women (34.2%) were exposed to HPV infection, with the highest overall infection rate (49.9%) among women aged 18 to 30 years (ie, early adulthood). These changes highlight the need for increased awareness among young people regarding the prevention of HPV infection. The average age at the time of the first instance of sexual intercourse among middle and high-school students in Korea has been reported to be 13.2 years, with the rate of sexual experience increasing every year by 5.9% [4]. Thus, educating adolescents about HPV and instilling awareness of early screening is critical.

In 2016, HPV vaccination became mandatory in South Korea, and the Healthy Women First Step Clinic project, providing free vaccines for girls aged ≥12 years, has been promoted [5]. The primary vaccination rate has increased since the implementation of the vaccination program in 2016. In 2017, it was 72.8% for those born in 2004 and 64.4% for the second dose [6], which was lower than the vaccination rates for the other 16 free vaccines mandated for Korean children below the age of 12 years (95% as of 2018) [6]. These suggest that interventions are needed to increase the willingness to be vaccinated against HPV at an age eligible for free vaccination.

Previous research reported a lack of education to alleviate HPV vaccine hesitancy among adolescents and insufficient communication regarding the positive effects of the vaccine [7]. The implementation rates of school health education programs regarding HPV vaccination among adolescents have been reported to be as low as 15% in South Korea, and health teachers’ accurate responses to questions related to HPV vaccine knowledge among health teachers was <77% [7]. These results suggest that school health education programs do not provide sufficient information on the HPV vaccine to middle-school girls who are at an appropriate age for HPV vaccination and that effective education programs and guidance on HPV vaccination for middle-school girls are needed. Thus, additional programs of age-appropriate vaccination initiatives addressing HPV infection prevention should be provided to middle-school girls aged 12 to 13 years, who are physically, cognitively, and psychologically able to absorb diverse knowledge and experience [8].

eHealth communication [9], which is designed to promote health through digital technologies, such as mobile and social media, has been used as an effective intervention strategy for the practice of adolescent health behaviors, particularly preventive health behaviors [10], HPV vaccination [11,12], prevention of binge drinking [13], and prevention of smoking [14]. Recently, developing a social networking service (SNS) platform that facilitates lasting relationships among its members and provides motivation and professional advice has attracted attention among health researchers [15]. Furthermore, the number of health chatbots that can provide interactive services for teenagers has increased [16,17]. It is important to strategically design eHealth communication that complements other health communication channels and facilitates easy-to-use and effective communication between health care professionals and patients [18]. In particular, mothers must be included in the health communication channel for HPV vaccination because mothers influence their daughters' vaccination decisions and serve as key health care decision-makers [19,20].

The rate of smartphone ownership among middle-school students in South Korea is 95.9%, the highest among adolescents [18], and communication apps represent a major health communication channel for disseminating health information to adolescents [19].

### Objective

Therefore, to increase HPV vaccination rates among middle-school girls, this study aimed to develop an eHealth communication interventions targeting middle-school girls and their mothers.

## Methods

### Overview

The eHealth communication intervention to promote HPV vaccination among middle-school girls in this study was developed using intervention mapping (IM) [21], a systematic approach to developing interventions using a detailed needs analysis of the subject. This paper describes the process of development and usability test of an eHealth communication intervention for middle-school girls and their mothers using SNS to improve HPV vaccination among middle-school girls. The intervention was developed in 6 steps: a needs assessment, setting the program outcomes and objectives, selecting a theory-based method and practical strategies, developing an intervention, developing an intervention plan, and testing the intervention.

### Step 1: Needs Assessment

#### Literature Review

Effective messages are important for health communication to influence the attitudes and behaviors of target groups. We searched the PubMed database for interventional studies related to health communication on HPV vaccination published after 2010. We then reviewed relevant studies that were published in English in international peer-reviewed journals between January 2010 and June 2020 to explore existing interventions involving the application of health communication on HPV vaccinations among adolescents. On the basis of PICO-SD (population, intervention, comparisons, outcomes-study design), the main research participants (P) of the literature were adolescents, and the intervention (I) included health communication for HPV vaccination of adolescents targeting adolescents, parents, and the community. For the comparison group (C), all studies on interventions targeting both nonintervention and nontreatment groups were included in the selection criteria. The intervention outcome (O) was adolescents’ HPV vaccination uptake and intention and their HPV knowledge. The study design included randomized control experimental, quasi-experimental, and single-group before-and-after designs. Studies were drawn from PubMed, CINAHL, Cochrane, and PsycINFO databases. Medical subject headings were used to select search terms. Frequent keywords were identified by analyzing English titles and keywords from the literature. The search strings were as follows: (((((“health communication” [Title/Abstract]) OR (“health message”[Title/Abstract])) AND (“HPV vaccin*” [Title/Abstract])) OR (“Human papillomavirus” [Title/Abstract])) AND (“program” [Title/Abstract] OR “intervention” [Title/Abstract] OR “campaign” [Title/Abstract])) AND (“adolescent” [Title/Abstract] OR “youth” [Title/Abstract] OR “teen*” [Title/Abstract]) (Multimedia Appendix 1).

**Figure 1:**
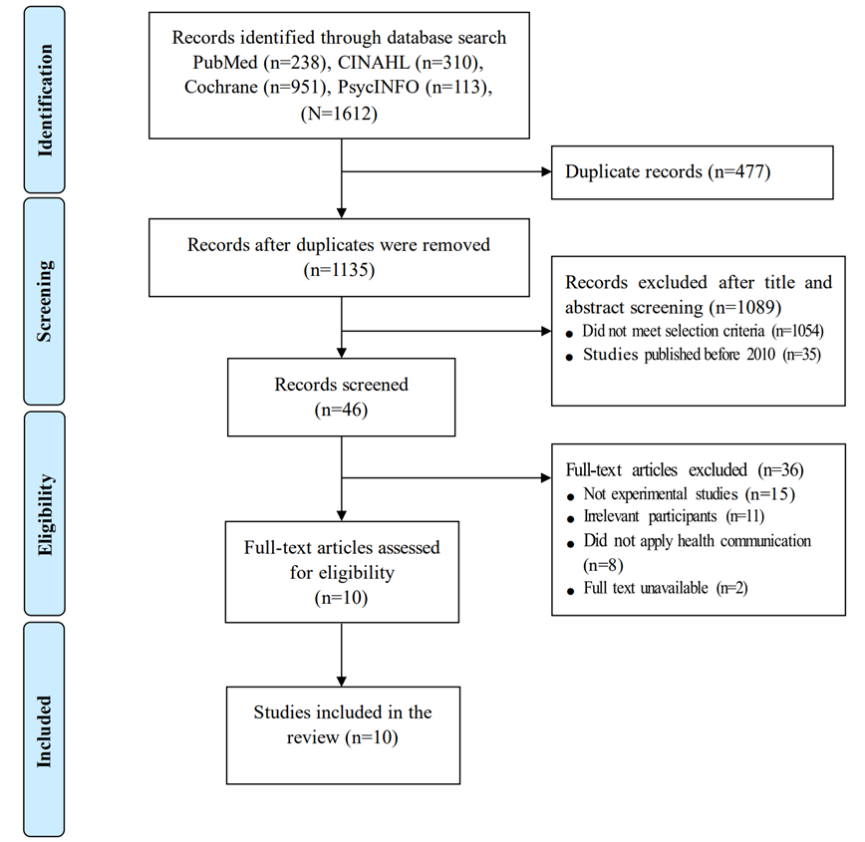
Flow diagram for study selection.

A bibliographic management program (EndNote X9; Clarivate) was used to sort and review studies retrieved from the database. The titles, abstracts, and full texts of all studies were reviewed in stages based on the selection and exclusion criteria. A total of 1612 studies were obtained from the 4 databases. Of these, 477 duplicate studies and 1089 studies with publication periods exceeding 10 years did not meet the selection criteria. A further 36 studies were excluded based on the exclusion criteria (Figure 1). Finally, by analyzing the 10 selected studies, the period, duration, and effect of intervention; design and content of health communication messages; theoretical framework; and delivery method were confirmed.

#### Focus Group Interview

A researcher recruited participants for focus group interviews (FGIs) from one school, which was different from the school recruiting the experimental group sample. The researcher explained the study’s purpose and contents to the health teachers and asked for cooperation. The school health teachers posted the recruitment notice on the school website and delivered the list of girls who expressed their intention to the researcher. A few participants were recruited through the snowball method, in which the interviewees recruited their friends from the same grade. Finally, 12 participants were recruited from adolescent girls who had received the second dose of the HPV vaccine, understood the purpose of the study, and obtained parental consent to participate in FGIs. The mean age of the 12 FGI participants was 15.45 (SD 0.52). Krueger and Casey [22] mentioned that small sample interviews with 4-6 people preferred participants to share their personal experiences freely. Therefore, we divided the 12 girls into 3 focus groups that included 4 participants. Following the guidelines published by Krueger [23], the questions were divided into 4 types, including introductory, transition, key, and closing questions (Textbox 1).

**Questions used in the focus group interviews.**

**Introduction**

- This is a comfortable interview to discuss your experiences and opinions to encourage human papillomavirus (HPV) vaccination, the cervical cancer prevention method. How old are you?

**Transition**

- You have completed the second dose of HPV vaccination, so when was the second dose vaccination?

**Key question 1**

- Knowledge: have you ever heard of cervical cancer and HPV? If you know about cervical cancer or HPV, please tell me.

**Key question 2**

- Perceived threat: do you consider yourself at higher risk of developing cervical cancer or HPV infection than someone else? Why do you think so? Have you ever known someone with cervical cancer or an HPV infection? Do you know the risk factors for HPV infection?

**Key question 3**

- Perceived barriers: what, if any, was the reason for your hesitancy to get vaccinated against HPV?

**Key question 4**

- Self-efficacy: what methods are needed for middle-school girls to overcome these barriers and start the vaccine uptake and complete the second dose?

**Key question 5**

- Cue to action: what strategies are effective for initiating and completing HPV vaccination? What incentives were middle-school students provided to participate in activities that encourage HPV vaccination?

**Closing**

- Please let us know if you have any additional comments you would like to share with unvaccinated students.

#### Data Analysis

The data collected during FGIs were systematically reduced in content through a qualitative content analysis. Meaningful interpretations were confirmed from the derived themes [24]. Direct content analysis, a type of qualitative content analysis, can enhance the validity of existing theories and expand the theoretical framework [25] and can be used to expand and supplement meaning by applying it a new research phenomenon when an existing theory on the phenomenon needed to be studied and follows a deductive structure [26].

Data analysis was conducted in 3 steps using the deductive approach suggested by Elo and Kyngäs [26]. First, we sought to understand the content by repeatedly reading the transcribed FGIs draft, treating each sentence as a unit for analysis. Second, we created categories and categorized corresponding sentences using a structured analysis approach based on the main concepts of the health belief model (HBM). Third, we further described and classified the data into the final themes and subthemes. To enhance the rigor of this study, credibility, neutrality, and consistency were applied to the data. At the end of the interview, the study participants’ responses were reviewed to increase their internal validity and truth value. To maintain neutrality, individual prejudices and judgments of the study and participants were excluded, and the content of the analysis and results were derived according to a qualitative content analysis procedure. The consistency of the study was improved by being verified by a nursing professor who had experience in several qualitative studies and did not participate in this study.

### Step 2: Setting Program Outcomes and Objectives

The second stage of IM specifies the intervention goals that will change because of the intervention. The goals are divided into intervention goals, expected outcomes, and specific performance objectives [21]. The final health outcome for the intervention was “Increase HPV vaccination uptake among middle-school girls.” The specific performance objectives were to identify positive changes in the key variables (ie, HPV vaccination knowledge, HPV vaccination health beliefs and responsibility, and sexual communication) and develop detailed performance objectives leading a final health outcome.

### Step 3: Selection of the Theoretical Method and Strategies

**Figure 2:**
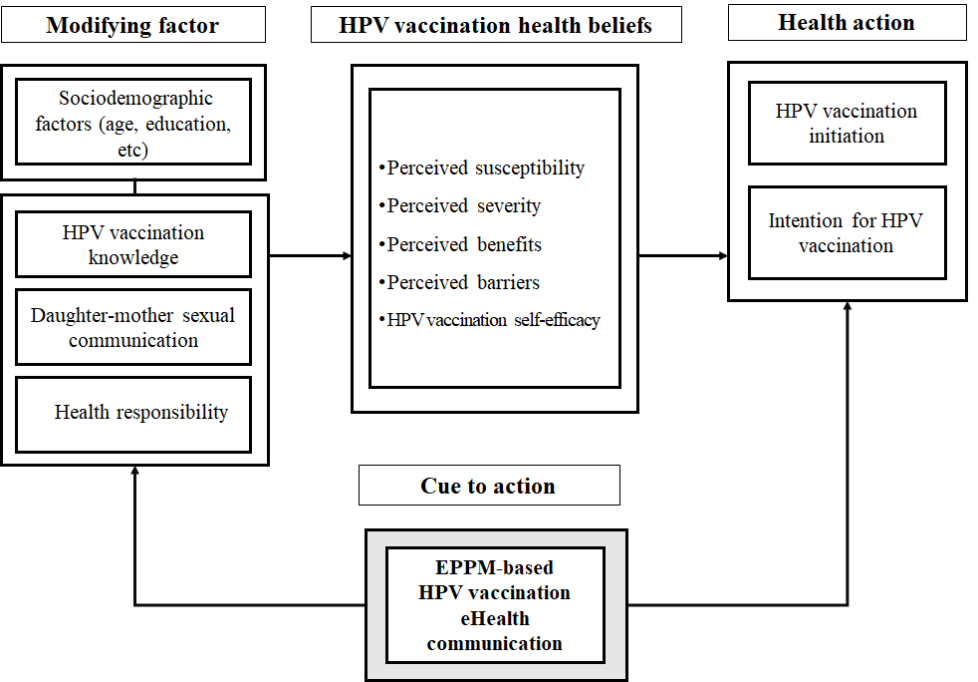
Conceptual framework of this study. EPPM: expanded parallel process model; HPV: human papillomavirus.

In the third step of IM, a theory and an appropriate method for studying changes in the related factors were developed. For the conceptual framework of this study, the HBM proposed by Champion and Skinner [27], which was modified by dividing the HBM proposed by Rosenstock [28] into modifiable factors and individual beliefs, was used. In addition, in this study, the expanded parallel process model (EPPM) proposed by Witte [29], a prominent theory in health communication research, was applied to construct an eHealth communication intervention. For the messaging to be successful, it is important to emphasize self-efficacy and high awareness of the perceived threat [30] and response efficacy (ie, the ability to avoid a threat if one takes the action recommended by the persuasion message)—in this case, the intention to receive the HPV vaccination. On the basis of a previous study [22] in which response efficacy had a significant effect on HPV vaccination intention rather than disease threat, the concept of EPPM was applied to the composition of eHealth communication. This conceptual framework is shown in Figure 2.

In addition, the themes derived from the FGIs and using the behavior change techniques (BCTs) [31] were included as specific intervention strategies to improve health beliefs about HPV vaccination. An effective BCT was selected by referring to experimental studies [32,33]. BCTs were used to increase vaccination intention and implementation.

### Step 4: Development of the Intervention

The intervention was designed using a theoretical framework based on the results of a literature review and FGIs and had a health communication strategy aiming to increase HPV vaccination rates among middle-school girls.

“Kakao i Open Builder,” an artificial intelligence (AI) platform developed by KakaoTalk, was used to deliver the HPV vaccination health communication through a chatbot (ie, software programs that can respond to text or voice messages).

In the first stage, the researcher had 3 face-to-face consultations with an expert who had the capacity to develop chatbots in the health and education fields using KakaoTalk to supplement the chatbot’s functions. The researcher also received advice from an expert through email communications and Zoom (Zoom Video Communications, Qumu Corporation) meetings during the development process. The researchers and experts discussed the types of chatbots that should be developed to improve HPV vaccination among middle-school girls. In the second step, the researchers designed a scenario consisting of questions based on the answers of the target group. In this step, we prepared questions and answers based on the details related to HPV vaccination (ie, cervical cancer, HPV, vaccine information, etc). Representative and similar words from frequently used terms were arranged. In the third stage, the implementation of the chatbot, written questions and answers were entered as utterance patterns and responses, and “skills” (ie, techniques that expresses a response to a user according to a topic, and it analyzes a question and creates an appropriate response) were developed so that answers matching key terms could be generated, and were connected with Open Builder. “Skill server” means receiving a request from a bot system, analyzing the information contained therein and responding appropriately. Images and videos necessary for health communication were developed based on the needs assessment. In the fourth stage, the chatbot was connected to the KakaoTalk channel for testing. The experts and researchers continuously tested and updated the chatbot. Additional block updates, troubleshooting, and maintenance were performed to address errors before the intervention was delivered.

### Step 5: Planning the Implementation of the Intervention

In the next stage, a plan was established to implement an eHealth communication intervention. Appropriate methods to recruit participants effectively and implement specific interventions during the COVID-19 pandemic were discussed by consulting with participating middle-school health teachers. Monitoring methods were established to control the quality of the intervention so that records, such as chatbot use frequency and commonly searched blocks, were collected through the KakaoTalk channel manager system.

### Step 6: Evaluation of the Validity of the Intervention

In the final stage, structured questionnaires, namely the Chatbot Usability Questionnaire (CUQ) [34] and Health Communication Message Review Criteria [35]**,** were used to evaluate the validity of the eHealth communication intervention. The CUQ and health message evaluation tool were translated from English to Korean, and the validity based on the expert and participant groups and the feasibility of the participants of the FGIs were evaluated. The CUQ comprises 16 items (eg, clear indication, error handling, user input language recognition, complexity, and convenience) that were evaluated on a 5-point Likert scale.

The health communication message review criteria comprised 12 minimum standards for a persuasive message and evaluated message clarity, evidence, reliability of delivery method, appropriateness of tone, and uniqueness of the message on a 4-point Likert scale. Because the original text of the CUQ and health communication message evaluation was in English, approval was obtained from the original author for translation and use. The committee approach to translation attempts to reduce the cultural bias inherent in the native language by striving for a consensus translation at the beginning of the translation process [36]. This contrasts with back-translation, in which a single individual initially translates, and then a panel of experts translates. In the committee approach, several translators individually translated the original questionnaire [37]. Therefore, in this study, the scales were translated independently by 2 PhD nursing students, including the researcher, who is fluent in both English and South Korean and has experience translating scales. The next step was for the committee of reviewers to review the translations and reach a preliminary consensus. In this study, 2 nursing professors fluent in English and South Korea were assigned as reviewers and attempted to reach a consensus on the translation of all questionnaire items. Finally, the accuracy of the translation into South Korea was verified by a PhD student from the Department of South Korean Language and Literature.

The prototype chatbot was validated by 3 nursing professors and 4 school health teachers using the translated CUQ and health communication message review criteria. Its feasibility was evaluated by 5 middle-school girls who participated in the FGIs also using the CUQ and health communication message review criteria. According to the method developed by Lynn [38], health message evaluation calculated the item content validity index (I-CVI) for each item, and items with an I-CVI of <0.80 were reviewed and corrected. The item “Message uses an appeal method suitable for the subject” had an I-CVI of 0.71, so it was reviewed and corrected. For the chatbot usability test, the average score was calculated using CUQ’s calculation tool. El Hefny et al [39] used the same tool to evaluate the usefulness of chatbots and set the user acceptance score at 71.1.

### Ethical Considerations

This study was approved by the Yonsei Health System Institutional Review Board before data collection (approval number Y-2020-0172). In this study, an electronic signature service called MODUSIGN was used instead of a paper signature. MODUSIGN is an electronic contract service in which a signature has legal effect. Before the study was conducted, all participants were given an explanation and a consent form through MODUSIGN. They signed the consent form on the web and participated in the study.

## Results

**Figure 3:**
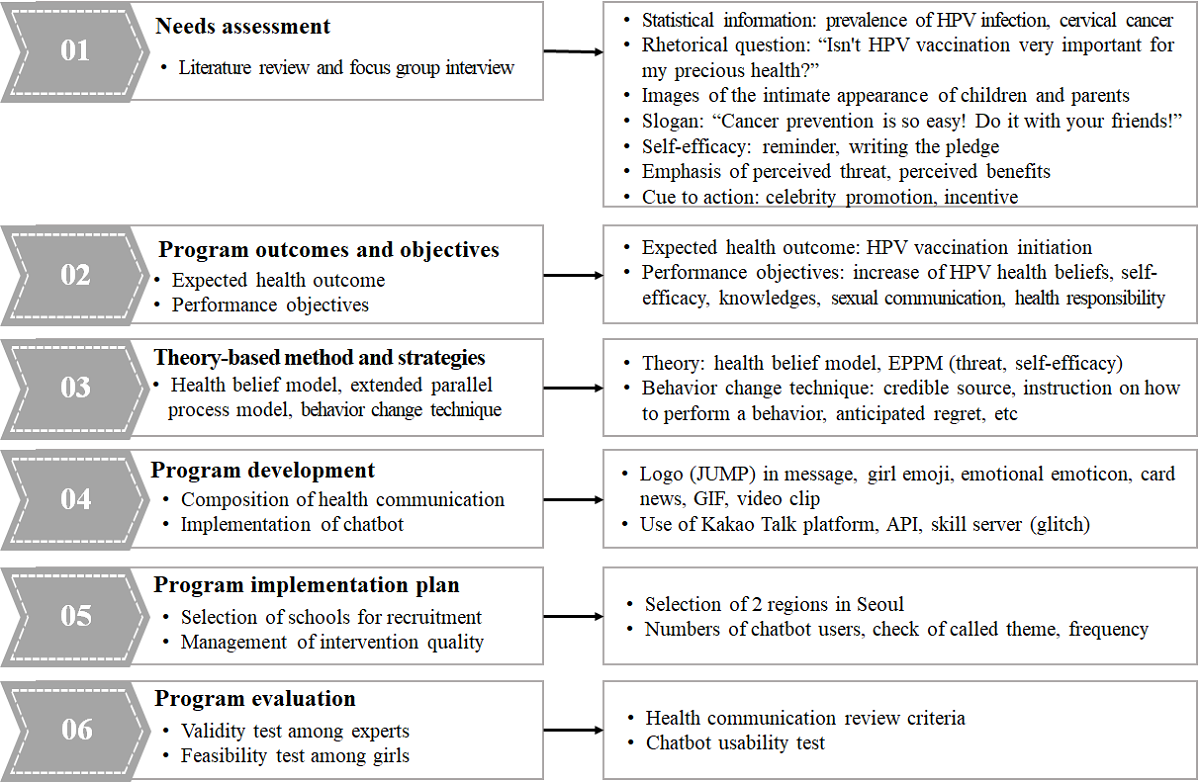
eHealth communication intervention development and adaptation process. EPPM: expanded parallel process model; HPV: human papillomavirus.

The eHealth communication intervention for HPV vaccination was developed using a 6-step IM process (Figure 3).

### Step 1: Needs Assessment

#### Literature Review

Overall, 10 health communication intervention studies on HPV vaccination among adolescents were examined to evaluate the effectiveness of health communication strategies in raising awareness about HPV vaccination and encouraging vaccination among this age group.

Half of the studies (5/10, 50%) used randomized controlled experimental designs, whereas the other half (5/10, 50%) used quasi-experimental designs. Of 10 studies, 4 (40%) included adolescents, 2 (20%) parents, 1 (10%) study parent, and 1 (10%) health professional, and 3 (30%) studies included local communities, including medical institutions (30%). The intervention duration ranged from a one-time engagement to 39 months.

As a characteristic of the health communication intervention program, a health communication message related to HPV vaccination was developed based on the main concepts of the HBM. The information included statistics on the subject, a message based on the main concept of HBM, and an image representing the intimacy between a mother and daughter.

The design of health communication messages for adolescent HPV vaccination considers the following strategies: information based on rhetorical questions (“Do you want to protect your daughter from cervical cancer?” and “If there is a vaccine that can prevent cervical cancer, would you like to vaccinate your children?”) [40], including precise figures for the disease and vaccine, messages based on key concepts from theory, posters using models (parents and children), and slogans.

Various intervention delivery methods were used in 100% (10/10) of the studies, including community social marketing campaigns through leaflets and posters, face-to-face education, mail, interviews, and web-based platforms such as SNS. A mixed method using leaflets, posters, and web-based media was used. eHealth communication using SNSs has been used to check the rapid spread of messages and real-time responses.

To improve HPV vaccination rates among adolescents, health communication strategies can rely on various channels, including web-based platforms, and improve confidence in the safety of vaccines among target groups.

#### Focus Group Interviews

From the FGIs, 5 categories and 10 themes corresponding to knowledge, perceived threats and barriers, self-efficacy, and cues-to-action were derived (Textbox 2). FGIs showed that the participants had low HPV vaccination knowledge, low perceived susceptibility and severity before taking the HPV vaccine, and various barriers to vaccination such as pain, side effects, and difficulty of receiving 2 doses. To overcome the barriers to vaccination and enhance vaccination intentions, detailed explanations from health care providers and school health teachers and recommendations for vaccination through various channels are necessary.

**Results of focus group interview.**

**Cervical cancer and human papillomavirus (HPV) infection knowledge**

- Low knowledge of cervical cancer and HPV infection
  Lack of cervical cancer knowledge
  Lack of HPV infection knowledge
  Lack of access to information

**Perceived threats**

- Low perceived susceptibility and sensitivity regarding cervical cancer and HPV
  Low susceptibility to cervical cancer and HPV infection
  Positive prejudice toward HPV infection

**Perceived barriers**

- Injection pain
  Threat of injection pain
- Side effects of HPV vaccination
  Threat of side effects
- The hassle of requiring 2 vaccinations
  Hassle of repeated vaccination
  Delayed vaccination due to missed timing

**HPV vaccination self-efficacy**

- Vaccination reminders
  Providing notices for repeated vaccinations
- Enhancing vaccination benefits
  The only cancer that can be prevented
  Best way to prevent cancer for free
  Cancer prevention health care through vaccination

**Cue to action**

- Promotion of vaccination through various channels
  Health care providers’ recommendations for vaccination
  Detailed explanation about the vaccine in school newsletters
  Participation in mobile activities about vaccination promotion
  Vaccination promotion on social networking service
  Vaccination promotion by celebrity
- Adolescent-friendly messaging
  Use of visual materials, such as emoticons, videos, and images
  Use of high chroma images
- Appropriate incentives
  Complimentary messages
  Material incentives

### Composing Health Communication for Vaccination

It was necessary to use messages that girls could understand in a friendly tone, accompanied by images or characters to enhance their understanding. It was also found that interactions could occur through mobile phones. In addition, while emphasizing the need for vaccination and positive awareness of vaccination in the message, intervention strategies, reminders to complete vaccination, and activities that encourage confidence in girls to decide and get vaccinated themselves, such as quizzes, providing incentives, and emotional support, were confirmed. Middle-school girls suggested using mobile phones to highlight the benefits of vaccination, such as free cancer prevention and health care, to middle-school girls and their mothers, and to exchange questions and answers in real time. They also mentioned that promoting vaccination on SNS and having celebrities endorse it would increase their interest.

### Step 2: Setting Program Outcomes

The expected behavioral outcome of the eHealth communication intervention was that middle-school girls will receive their first dose of the HPV vaccine within 2 months of participation in the intervention. On the basis of the theoretical framework and the HBM, the change determinants were set as health beliefs, self-efficacy, knowledge, sexual communication, and health responsibility. With these changes, the following 2 performance objectives focused on the behavior change of middle-school girls were composed: “I will vaccinate myself to prevent HPV infection” and “I will look up information on HPV vaccination through the chatbot.”

### Step 3: Selection of a Theory-Based Method and Practical Strategies

The HBM proposed by Champion and Skinner [27] shows that modifiable factors (eg, age, education level, race, and knowledge) can affect an individual’s health beliefs and that an individual’s health beliefs directly affect preventive health behaviors. Of these modifiable factors, health education is the most necessary before other interventions to promote the prevention behaviors of HPV infection [41] because an increase in knowledge through education increases preventive behavior [28].

In previous studies [42,43], the intention to change health behavior to prevent HPV infection and cervical cancer improved after educating the patients about cervical cancer and HPV infection. Therefore, knowledge appears to be critical for promoting preventive health behaviors. HPV infection prevention can also be included in sexual and reproductive health promotion practices because HPV infection is transmitted through sexual contact. A meta-analysis [44] examining the effects of parent-child sexual communication on adolescents’ safe sexual behavior found that sexual communication with mothers resulted in a safer sexual behavior, particularly among girls. Adolescents face the challenge of transitioning from health care dependent on parents to personal responsibility for health; thus, they need opportunities to develop and express their health care autonomy [45]. Information delivery through outlets such as mass media acts as cues for action urging individuals to engage actively in preventive health behavior and is important in determining health behavior [46]. This study set the eHealth communication intervention as the HPV vaccination uptake “cues for action.” Therefore, the HBM was established as the study’s theoretical framework, and sexual communication between parents and children and health responsibility of middle-school girls were set as additional modifying factors affecting HPV vaccination uptake.

Practical strategies for designing the eHealth communication intervention were constructed based on BCT, following the results of the literature review and needs assessment (Table 1). The contents to improve HPV vaccine knowledge consisted of images, texts, and quizzes.

**Table 1:** eHealth communication concepts, contents, theories, and methods.

Concepts	Contents	Behavior change techniques	Methods
Knowledge	Information about cervical cancer and HPVa Types, number of times, and process of HPV vaccination Information about vaccinations from medical institutions	Credible sources Information about health consequences Instruction on how to perform a behavior Material incentives Social rewards	Images, text, weblink, quizzes, and counseling
Sexual communication	Communication skills with mothers Practicing sexual communication	Credible sources Social support (emotional and practical) Instruction on how to perform a behavior Feedback on behavior	Images, text, weblinks, and activity for mothers and children to talk about sexual health
Health responsibility	Encouragement to seek health information Induction of HPV vaccination as a form of health care	Social support (practical) Comparative imagining of future outcomes	Images, text, and counseling
Perceived threats	Causes, symptoms, and prevalence of HPV infection Causes, symptoms, and prevalence of cervical cancer	Information about health consequences Credible sources Pros and cons Comparative imagining of future outcomes Anticipated regret	Images, text, quizzes, and self-examinations
Perceived benefits	Effects of HPV vaccine (domestic and international)	Credible sources Information about health consequences Pros and cons Salience of consequences	Images, text, quizzes, and celebrity endorsements video
Perceived barriers	Misunderstandings about HPV vaccine Coping with side effects Rules for vaccination	Credible sources Information about health consequences Information about antecedents Instruction on how to perform a behavior	Images, text, quiz, weblink, and phone calls
Self-efficacy	Setting of second HPV vaccination notification Writing a pledge to be fully vaccinated	Self-monitoring of behavior Behavioral contract Action planning Self-talk	Images, video text, and activities
Cues for action	Providing praise and incentives for participating in quizzes Providing praise and incentives for vaccination initiation	Social rewards Material incentives	Images and incentives

^a^HPV: human papillomavirus.

According to the HBM, the “threat” should be explained in terms of perceived susceptibility and severity. The intervention was designed to explain the severity of the disease caused by HPV infection and that anyone could easily get it, and to check the risk of HPV infection. We provided card news (a new concept news report optimized for mobile environments created by combining images and text) about a woman diagnosed with cervical cancer before marriage because she had not been vaccinated to convey the importance of getting vaccinated and the seriousness of the disease. The perceived benefits were conveyed through images and text that explained that the HPV vaccine is effective in preventing cervical cancer, the benefits of vaccination have been proven, and the HPV vaccine was included in the National Immunization Program worldwide. Perceived barriers were addressed through information that dispelled misunderstandings about vaccinations, side effects, and precautions, was communicated through images and text, and connected to the Korea Centers for Disease Control and Prevention (KDCA) call center to report the side effects of the HPV vaccine. The researcher provided reminders, encouraged the girls to take a pledge to get vaccinated, and developed animation about HPV vaccination support to overcome vaccinations barriers and improve participants’ self-efficacy. The “cues to action” were to increase positive awareness and participants’ intentions through eHealth communication intervention and to provide praise and material incentives, referring to the results of the needs assessment and the BCT of “social reward” and “material incentive.”

### Step 4: Development of the eHealth Communication Intervention

#### Implementation of the Chatbot

**Figure 4:**
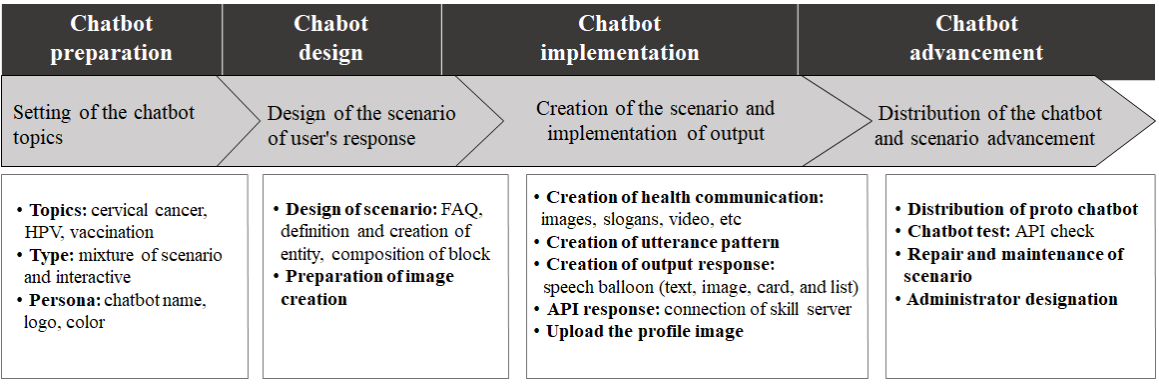
Process map of chatbot development. API: application programming interface; FAQ: frequently asked questions.

A chatbot was developed over 4 steps: preparation, design, implementation, and advancement (Figure 4).

In the first step of the chatbot development process, the chatbot’s topic was set and was selected as a mix of a “scenario type” (ie, providing responses according to set scenarios) and a “conversation type” (ie, answering questions when keywords are input). The chatbot’s “persona” was its overall impression and corresponded to its profile image and tone [47]. It was named “JUMP” (Junior Vaccine Uptake through an M-health Program). JUMP aimed to convey the ideas of being “vigorous,” “dynamic,” “bright,” and “healthy” as its persona and the attributes defining its social role.

In terms of chatbot design, it was set with images that showed a smart girl wearing a school uniform character who provided the overall message of the chatbot and evidence-based health information to give middle-school girls the sense of a peer’s friendliness. Among the 5 personality types (ie, extroverted, agreeable, conscientious, neurotic, and open) identified [48], JUMP’s personality was classified as agreeable and conscientious. On the basis of the input from the FGIs, the chatbot’s logo was designed in shades of orange, and its interface was designed in yellow and pink to convey brightness and warmth.

In the second step of the chatbot development process, information about cervical cancer, HPV infection, and vaccination was obtained from credible sources, including the KDCA and the National Cancer Information Center. Information about cervical cancer and HPV was organized in a question and answer format in an Excel (Microsoft Corporation) file to design the chatbot scenarios. Next, an “Entity” was defined, which was a data dictionary that systematically organized terms that the chatbot can understand. The type of “Entity” was set to be linked to the subject, and the data dictionary was built by entering cancer and virus terms (eg, *HPV*, *Human papillomavirus*, *human papillomavirus*, *Human papilloma virus*, and *hpv*) and vaccine terms (eg, *vaccine*, *vaccination type*, *injection*, *Gardasil*, and *Cervarix*).

In the third step of the chatbot development process, the “utterance pattern” and “responses” were set. The chatbot can select and show the block corresponding to each “intent” by registering the utterance pattern even if the user speaks slightly differently. A logo and image designed to appear friendly to middle-school students were created based on the FGIs, and health information, statistics, and slogans were included in the image to clarify the meaning of health communication and to make it easier for users to understand the intention of the message. In addition to the researcher’s data, card news distributed by the KDCA was used as visual data. The results of the FGIs indicated that middle-school girls had a low awareness of HPV, so a generic menu was implemented with the topic (block) provided by the chatbot immediately upon entering rather than using a typing-oriented chatbot. The connections between the detailed blocks according to the generic menu are shown in Multimedia Appendix 2. The menu was in the form of a slider at the bottom of the chatbot window. When the user swiped the screen upward, all essential blocks appeared. Each block in the generic menu had a speech bubble of its own, followed by a carousel in which information was displayed. The user’s utterance was input for each block, so that when the chatbot recognized the user’s question, a detailed response was presented. The response setting used a mixture of text (1000 characters), images, cards (image and text, up to 40 characters), and lists (up to 5), with the help of various speech bubble functions on KakaoTalk. The direct link function helps users navigate the chatbot. Websites such as the KDCA or YouTube videos were linked with messages provided by the chatbot to provide users with health information on cervical cancer and HPV vaccinations. When participants did not receive responses to their questions from the chatbot, the 1:1 chat function was available to enable the researcher to respond to any chatbot errors by answering the participants’ questions in real time. To deliver dynamic data to users, the Glitch server was linked with Open Builder so that questions and answers could be output through an application programming interface. The completed chatbot was named JUMP 2021, and an image of the JUMP logo was uploaded to the profile.

The fourth stage was the advancement stage of the chatbot involving the distribution of the chatbot by the researcher and developer, its experimental use, and updating of additional necessary content. We were set as administrators within Open Builder to resolve errors to responses that were not output through the skill data, to continuously maintain and repair the JUMP chatbot during the intervention period.

#### Development of Health Communication Messages

Health communication messages about HPV vaccination were developed based on 12 review criteria [35] that can be used in both the pre- and posttesting stages to evaluate health communication messages and campaigns.

The health communication messages included information about cervical cancer, HPV infection and vaccination, information about neighborhood vaccination sites, social support for HPV vaccination, and questions and answers.

The JUMP logo was inserted at the top of each message to show the uniqueness (identity) of the health communication message. Reliable public institutions such as the KDCA were cited when providing information, statistics, and explanations, as well as prevention guidance and statistics about diseases of public interest. The data sources were included for each health message. Reflecting on FGIs results, a female student explained health messages to provide a friendly feeling. Emojis are used to make health messages more engaging and easily understandable. Messages were conveyed professionally and credibly. To improve participants’ self-efficacy regarding vaccination and address the burden identified by FGIs, chatbot messaging (in the form of images and text) highlighted that only 2 HPV vaccinations were required and administered at 6-month intervals. In addition, a self-checklist for cervical cancer risk published by the American Cancer Society was provided to improve self-efficacy and raise awareness on the susceptibility and severity of cervical cancer. Emotional support was provided to the respondents through emojis or 1:1 chatting to encourage their health and health information-seeking behaviors.

**Figure 5:**
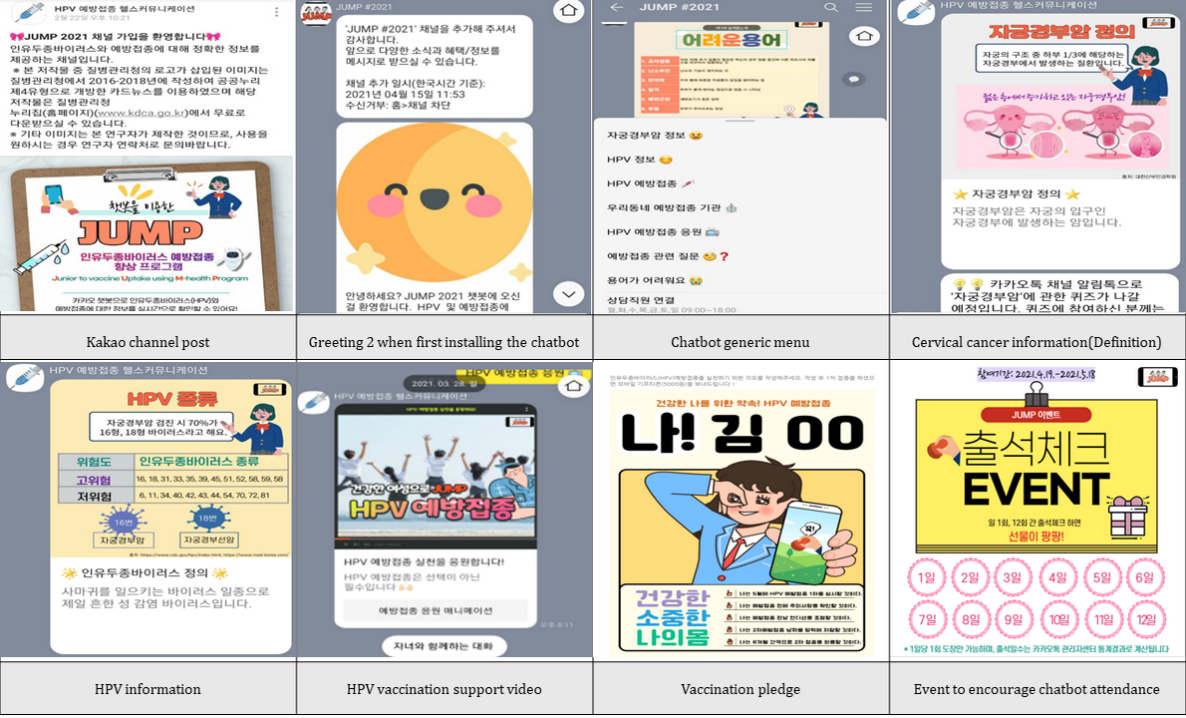
Screenshot of JUMP (Junior Vaccine Uptake through an M-health Program) eHealth communication.

In this study, the eHealth communication intervention to be provided to the experimental group was planned. Four additional activities were designed to conduct in the experimental group to increase awareness of HPV vaccination, enhance vaccination intention, and promote participation in eHealth communication. In the first week, a quiz on cervical cancer and HPV infection was administered. In the second week, an HPV vaccination quiz was administered. In the third week, the participants were invited to pledge to be vaccinated against HPV infection. In the fourth week, sexual communication between mothers and daughters regarding appropriate sexual health is recommended. Screenshots of the JUMP chatbot intervention are presented in Figure 5.

### Step 5: Implementation Plan

The researcher visited the experimental and control groups and explained the study to the school health teachers, the principal and vice principal, and received approval. Due to the COVID-19 pandemic, first-grade middle students were attending school irregularly at time of data collection, so it was decided that using a chatbot was an appropriate intervention method. The researcher asked the school health teachers to encourage participation and sought cooperation to prevent dropouts. A log recording system was built and monitored by the researcher with the help of a chatbot development expert that checked the frequency of block use and the content of the frequently used blocks for each participant to manage the quality of the intervention.

### Step 6: Testing the Validity of the Intervention

The validity of the eHealth communication intervention was evaluated by experts, and its feasibility was tested in a sample of middle-school girls. Three nursing professors with experience in developing interventions and 4 school health teachers currently managing HPV vaccination at the middle-school level were the experts that evaluated the usability of the chatbot and the content of the health communication strategy. The chatbot usability was evaluated on a 5-point Likert scale with 16 items with the scores being canulated according to the original measurement. The experts’ chatbot usability scores ranged from 67.19 to 90.63, with a mean of 80.13 (SD 8.15), which was higher than the minimum score (71.1) for chatbot acceptance of the study [39]. The supplemented content included “there are terms that are difficult for middle-school students to understand” and “there is a need to motivate adolescents to participate until the end.” A block explaining difficult terms was added to the chatbot to address these points, and biweekly notifications were provided to encourage continuous participation. From April 19 to May 18, 2021, if participants used any block more than once in the JUMP chatbot, it was counted as attendance. In this study, the implementation standard for intervention participation was set to at least 3 times a week of chatbot use based on a previous intervention study using a health chatbot [49]. The content validity of the health communication messages was evaluated by experts using a 4-point scale comprising 12 items. The average I-CVI was 0.94. Of the 12 items, 1 item had a score <0.8, indicating that “The message uses an appealing tone suitable for the target (ie, rational or emotion).” The tone of the messages was made more friendly by mixing praises and positive images to address this point and ensure that self-efficacy would increase instead of perceived threat.

Five students who participated in the FGIs and used the chatbot for 1 week immediately after joining the channel evaluated the chatbot intervention feasibility using the chatbot usability and health communication message measures. Their usability scores ranged from 73.44 to 92.19, with a mean score of 84.06 (SD 7.61), which was higher than the previously identified minimum score for chatbot acceptance [39]. According to the middle-school students, the advantages of the chatbot were “It is very intuitive and easy to use,” “It answers kindly and quickly,” and “It is easy to get an answer right away when you press a button.” The average I-CVI of the health communication message was 0.98. All items scored ≥0.8. The students shared that “The pictures are friendly,” “It’s good because it uses emoticons,” and “It’s good that the health information is detailed.” There were no additional corrections or supplements from the open-ended opinions on the usefulness of chatbots or the evaluation of health communication messages.

## Discussion

### Principal Findings

The eHealth communication intervention was developed by applying IM, a theoretical framework for developing programs aimed at changing health behaviors. The systematic process has been shown to be effective in integrating theoretical and empirical evidence and planning theory-based interventions. Several studies [50-52] have highlighted its application as an intervention development strategy for HPV vaccination among adolescents. In this study, IM guided the development of systematic interventions, such as designing health communication messages appropriate for middle-school girls and their mothers and deriving motivational strategies to participate in the intervention. This study is significant in that it was attempted as a youth-friendly intervention that connects AI and data in the era of the 4th industrial revolution and uses chatbot to deliver health communication messages.

Most existing interventions for adolescents or their parents have focused on providing information about cervical cancer, HPV infection, the effects and safety of vaccinations, and vaccination methods [53,54]. In this study, BCT was applied both to provide knowledge and strengthen the adolescents’ self-efficacy regarding vaccination and vaccination intention. Marshall et al [55] presented potential interventions using BCT to reduce HPV vaccine hesitancy, which was partially consistent with the BCT applied in this study. Examples of effective BCTs for HPV vaccination included “information about health consequences,” “salience of consequences,” “anticipated regret,” “credible source,” and “pros and cons” [55]. In addition, among the BCTs presented by Marshall et al [55], “identification of the self as a role model,” which encourages the person who has been vaccinated against HPV to set an example for others, should be used in future research. Because BCT has been used as a strategy to enhance both HPV vaccination rates and cervical cancer screening [56], it could be used as a practical strategy in future intervention studies to encourage cervical cancer screening among women in their twenties.

Designing a chatbot system tailored to address a target audience requires an understanding of the individual target users’ background, including their demographic characteristics, living environment, and personality [57]. Interventions tailored to suit these characteristics are effective because the behavior change strategy and persuasive messages can be adjusted according to the target group’s specific needs [51]. According to the behavior change model for health-related chatbots [57], the 4 necessary components included design of chatbot characteristics with a comprehensive understanding of the users’ backgrounds, the relational capabilities of the chatbot (eg, social dialogue and empathetic messages), persuasive conversation skills, and an evaluation method for both the user experience and health outcomes. This study’s chatbot was designed to display text-based health information on the HPV vaccine. In addition, based on the results of FGIs, the chatbot’s persona was designed to be youth-friendly and trustworthy to build the relational competency of the chatbot. Thus, this study can be used as a scientific basis for effectively designed chatbots. In the chatbot behavior change model, the components for persuasive dialogue capacity included rhetorical appeal, credibility (evidence of data), and specific persuasive messages (eg, self-efficacy enhancement narrative and case interviews). In this study, scientific evidence and credible data were presented to provide persuasive health communications about HPV vaccination. Rhetorical expressions (“Isn’t HPV vaccination very important to protect my priceless health?” “Vaccination is only two doses!”) and the case study of an unvaccinated woman diagnosed with cervical cancer were presented. Thus, the chatbot can be seen as an appropriate tool to motivate participants to change their behavior.

The credibility of a health communication intervention is important, particularly when it aims to ensure user acceptance. This study’s chatbot was designed to be youth-friendly, reliable, and professional. The credibility, professionalism, and authenticity of the messages are crucial, as they can influence the persuasiveness of health communication strategies [58,59], and misleading health information can lead to negative health outcomes or the failure to implement health behaviors [60]. Reliability can increase when the intention and expertise of the research team managing the chatbot are introduced to the participants [57]; therefore, in this study, the researcher’s affiliation and the JUMP logo were included in the notification messages, and the sources for all health communication information were provided. Previous research has reported that systematic health communication delivery through health professionals is important to provide accurate information about HPV vaccination in adolescents [61,62]; thus, the HPV vaccine communication strategy involved providing active recommendations for HPV vaccination by health care professionals. Therefore, in this study, the researcher, as a health communication channel, was expected to have a positive effect on the participants’ intention for HPV vaccination.

In this study, health communication was delivered through a chatbot, a cost-effective method of providing health information to adolescents [63]. It was reported that chatbots are useful for life-skill coaching among youth, are easy to use, and are interesting enough to encourage participation from peers [16]. Stephens et al [64] reported that using chatbots to promote weight management and care among +adolescent patients who were prediabetic was effective in treatment adherence, behavior change, and overall health promotion. Therefore, in this study, a Kakao Talk chatbot was developed as a delivery method for HPV vaccination health communication. This study can be used as an empirical support for nursing interventions involving social media and an AI platform considering the positive function of SNS with the advantages of interaction and convenience.

As chatbots improve their ability to understand user intent and respond to input, the amount of personal information shared in chatbots may increase. Therefore, future research should emphasize privacy protection from unauthorized users of chatbots [65]; identify privacy and data safeguards within the system; and provide specific explanations to audiences about privacy, data anonymization, and so on before deploying chatbots.

### Limitations

This study has several limitations. While developing the eHealth communication intervention for HPV vaccination, FGIs were conducted only with middle-school girls, the main targets of the intervention. Follow-up research should conduct FGIs with mothers and develop an intervention program suitable for mothers based on its results to supplement this study’s eHealth communication. In addition, the number of daily chatbot visits was not counted. Several visits within a single day using the same KakaoTalk profile were counted as one visit. It will be necessary to supplement the system to enable the calculation of repeated chatbot participation in the research design.

### Conclusions

This study developed and evaluated the usability of a health communication intervention to increase HPV vaccination rates among middle-school girls that was based on EPPM and the HBM and delivered via chatbot. The intervention targeted middle-school girls and their mothers who made decisions concerning adolescent health care. To boost the intention to continue using the JUMP chatbot, a periodic question and answer process should be updated to satisfy ongoing user intent and questions by monitoring participants’ responses. This study confirmed the usability of the health communication strategy to encourage HPV vaccination and is expected to contribute toward establishing a foundation for future youth-friendly health communication strategies.

## Appendix

Multimedia Appendix 1 Sample search strategy.

Multimedia Appendix 2 Generic menu of JUMP (Junior Vaccine Uptake through an M-health Program) chatbot.

## Abbreviations

AI: artificial intelligence
BCT: behavior change technique
CUQ: Chatbot Usability Questionnaire
EPPM: expanded parallel process model
FGI: focus group interview
HBM: health belief model
HPV: human papillomavirus
I-CVI: item content validity index
IM: intervention mapping
JUMP: Junior Vaccine Uptake through an M-health Program
KDCA: Korea Centers for Disease Control and Prevention
PICO-SD: population, intervention, comparisons, outcomes-study design
SNS: social networking service

## Data Availability

The datasets generated during or analyzed during this study are available from the corresponding author on reasonable request.

## References

[1] Press release. Health Insurance Review and Assessment Service.

[2] World Health Organization (2014). Human papillomavirus vaccines: WHO position paper, October 2014. Wkly Epidemiol Rec.

[3] Lee EH, Um TH, Chi HS, Hong YJ, Cha YJ (2012). Prevalence and distribution of human papillomavirus infection in Korean women as determined by restriction fragment mass polymorphism assay. J Korean Med Sci.

[4] Results announcement book. Korea National Health and Nutrition Survey.

[5] National children's immunization support project. Korea Disease Control and Prevention Agency.

[6] HPV National vaccination support project. Korea Disease Control and Prevention Agency.

[7] Kang M (2015). A survey of school nurses` knowledge on HPV vaccine and a status of educations about cervical cancer prevention. Health Nurs.

[8] Edelman CL, Kudzma EC, Mandle CL (2013). Health Promotion Throughout the Life Span.

[9] Kreps GL (2017). Ehealth communication. Oxford Research Encyclopedia of Communication.

[10] Badawy SM, Kuhns LM (2017). Texting and mobile phone app interventions for improving adherence to preventive behavior in adolescents: a systematic review. JMIR Mhealth Uhealth.

[11] Cates JR, Diehl SJ, Crandell JL, Coyne-Beasley T (2014). Intervention effects from a social marketing campaign to promote HPV vaccination in preteen boys. Vaccine.

[12] Dempsey AF, Pyrznawoski J, Lockhart S, Barnard J, Campagna EJ, Garrett K, Fisher A, Dickinson LM, O'Leary ST (2018). Effect of a health care professional communication training intervention on adolescent human papillomavirus vaccination: a cluster randomized clinical trial. JAMA Pediatr.

[13] Carrà G, Crocamo C, Bartoli F, Carretta D, Schivalocchi A, Bebbington PE, Clerici M (2016). Impact of a mobile e-health intervention on binge drinking in young people: the digital-alcohol risk alertness notifying network for adolescents and young adults project. J Adolesc Health.

[14] Khalil GE, Wang H, Calabro KS, Mitra N, Shegog R, Prokhorov AV (2017). From the experience of interactivity and entertainment to lower intention to smoke: a randomized controlled trial and path analysis of a web-based smoking prevention program for adolescents. J Med Internet Res.

[15] Piao M, Kim J, Ryu H, Lee H (2020). Development and usability evaluation of a healthy lifestyle coaching chatbot using a habit formation model. Healthc Inform Res.

[16] Gabrielli S, Rizzi S, Carbone S, Donisi V (2020). A chatbot-based coaching intervention for adolescents to promote life skills: pilot study. JMIR Hum Factors.

[17] Mariamo A, Temcheff CE, Léger PM, Senecal S, Lau MA (2021). Emotional reactions and likelihood of response to questions designed for a mental health chatbot among adolescents: experimental study. JMIR Hum Factors.

[18] Kreps GL, O'Hair HD, O'Hair MJ (2020). The emerging area of e-health communication research: using data to enhance the effectiveness of health information systems. The Handbook of Applied Communication Research.

[19] Damnjanović K, Graeber J, Ilić S, Lam WY, Lep Ž, Morales S, Pulkkinen T, Vingerhoets L (2018). Parental decision-making on childhood vaccination. Front Psychol.

[20] Kornides M, Head KJ, Feemster K, Zimet GD, Panozzo CA (2019). Associations between HPV vaccination among women and their 11-14-year-old children. Hum Vaccin Immunother.

[21] Bartholomew LK, Fernández ME, Parcel GS, Kok G, Gottlieb NH (2016). Planning Health Promotion Programs: An Intervention Mapping Approach.

[22] Krueger RA, Casey MA (2014). Focus Groups: A Practical Guide for Applied Research.

[23] Krueger RA (2002). Designing and conducting focus group interviews. University of Minnesota.

[24] Roller MR, Lavrakas PJ (2015). Applied Qualitative Research Design: A Total Quality Framework Approach.

[25] Hsieh HF, Shannon SE (2005). Three approaches to qualitative content analysis. Qual Health Res.

[26] Elo S, Kyngäs H (2008). The qualitative content analysis process. J Adv Nurs.

[27] Champion VL, Skinner CS, Glanz K, Rimer BK, Viswanath K (2008). The health belief model. Health Behavior and Health Education: Theory, Research, and Practice.

[28] Rosenstock IM (1974). Historical origins of the health belief model. Health Educ Monogr.

[29] Witte K (1992). Putting the fear back into fear appeals: the extended parallel process model. Commun Monogr.

[30] Seon-Jeong L, Su-Beom L (2016). Trends in health communication research: comparison of communication and public health/pharmaceutics: the current state of health communication research. Korean Advert PR Soc.

[31] Michie S, Richardson M, Johnston M, Abraham C, Francis J, Hardeman W, Eccles MP, Cane J, Wood CE (2013). The behavior change technique taxonomy (v1) of 93 hierarchically clustered techniques: building an international consensus for the reporting of behavior change interventions. Ann Behav Med.

[32] Borg K, Sutton K, Beasley M, Tull F, Faulkner N, Halliday J, Knott C, Bragge P (2018). Communication-based interventions for increasing influenza vaccination rates among aboriginal children: a randomised controlled trial. Vaccine.

[33] Williams L, Gallant AJ, Rasmussen S, Brown Nicholls LA, Cogan N, Deakin K, Young D, Flowers P (2020). Towards intervention development to increase the uptake of COVID-19 vaccination among those at high risk: outlining evidence-based and theoretically informed future intervention content. Br J Health Psychol.

[34] Holmes S, Moorhead A, Bond R, Zheng H, Coates V, McTear M (2019). Usability testing of a healthcare chatbot: can we use conventional methods to assess conversational user interfaces?. Proceedings of the 31st European Conference on Cognitive Ergonomics.

[35] (2002). Health communication message review criteria. The Health Communication Unit.

[36] Furukawa R, Driessnack M, Colclough Y (2014). A committee approach maintaining cultural originality in translation. Appl Nurs Res.

[37] Douglas SP, Craig Cs (2007). Collaborative and iterative translation: an alternative approach to back translation. J Int Market.

[38] Lynn MR (1986). Determination and quantification of content validity. Nurs Res.

[39] El Hefny W, Mansy Y, Abdallah M, Abdennadher S (2021). Jooka: a bilingual chatbot for university admission. Proceedings of the 9th World Conference on Information Systems and Technologies.

[40] Rickert VI, Auslander BA, Cox DS, Rosenthal SL, Rupp RE, Zimet GD (2015). School-based HPV immunization of young adolescents: effects of two brief health interventions. Hum Vaccin Immunother.

[41] Lee EJ, Kim HO (2011). [Effects of human papillomavirus vaccination education on college women's knowledge, health belief, and preventive behavior intention]. J Korean Acad Nurs.

[42] McPartland TS, Weaver BA, Lee SK, Koutsky LA (2005). Men's perceptions and knowledge of human papillomavirus (HPV) infection and cervical cancer. J Am Coll Health.

[43] Lopez R, McMahan S (2007). College women’s perception and knowledge of human papillomavirus (HPV) and cervical cancer. Calif J Health Promot.

[44] Widman L, Choukas-Bradley S, Noar SM, Nesi J, Garrett K (2016). Parent-adolescent sexual communication and adolescent safer sex behavior: a meta-analysis. JAMA Pediatr.

[45] Ayres CG, Pontes NM (2018). Use of theory to examine health responsibility in urban adolescents. J Pediatr Nurs.

[46] Lee JS, Sohn J (2008). Research on attitudes of Daejeon citizens toward the domestic adaption. J Korea Contents Assoc.

[47] Pricilla C, Pricilla DP, Dharma D (2018). Designing interaction for chatbot-based conversational commerce with user-centered design. Proceedings of the 5th International Conference on Advanced Informatics: Concept Theory and Applications.

[48] McCrae RR, John OP (1992). An introduction to the five-factor model and its applications. J Pers.

[49] Piao M, Ryu H, Lee H, Kim J (2020). Use of the healthy lifestyle coaching chatbot app to promote stair-climbing habits among office workers: exploratory randomized controlled trial. JMIR Mhealth Uhealth.

[50] Rodriguez SA, Roncancio AM, Savas LS, Lopez DM, Vernon SW, Fernandez ME (2018). Using intervention mapping to develop and adapt two educational interventions for parents to increase HPV vaccination among Hispanic adolescents. Front Public Health.

[51] Crawford CA, Shegog R, Savas LS, Frost EL, Healy CM, Coan SP, Gabay EK, Spinner SW, Vernon SW (2019). Using intervention mapping to develop an efficacious multicomponent systems-based intervention to increase human papillomavirus (HPV) vaccination in a large urban pediatric clinic network. J Appl Res Child.

[52] Austin JD, Rodriguez SA, Savas LS, Megdal T, Ramondetta L, Fernandez ME (2020). Using intervention mapping to develop a provider intervention to increase HPV vaccination in a federally qualified health center. Front Public Health.

[53] Choi JY, Choi SY (2013). Effects of human papilloma virus on related education for female high school students. Asian Oncol Nurs.

[54] Yeom YR, Lim SM (2019). Trends in domestic and foreign studies on the effect of preventing cervical cancer program in parents. J Korea Contents Assoc.

[55] Marshall S, Sahm LJ, Moore AC, Fleming A (2019). A systematic approach to map the adolescent human papillomavirus vaccine decision and identify intervention strategies to address vaccine hesitancy. Public Health.

[56] Nakisige C, Trawin J, Mitchell-Foster S, Payne BA, Rawat A, Mithani N, Amuge C, Pedersen H, Orem J, Smith L, Ogilvie G (2020). Integrated cervical cancer screening in Mayuge District Uganda (ASPIRE Mayuge): a pragmatic sequential cluster randomized trial protocol. BMC Public Health.

[57] Zhang J, Oh YJ, Lange P, Yu Z, Fukuoka Y (2020). Artificial intelligence chatbot behavior change model for designing artificial intelligence chatbots to promote physical activity and a healthy diet: viewpoint. J Med Internet Res.

[58] König L, Breves P (2021). Providing health information via Twitter: professional background and message style influence source trustworthiness, message credibility and behavioral intentions. J Sci Commun.

[59] König L, Jucks R (2020). Effects of positive language and profession on trustworthiness and credibility in online health advice: experimental study. J Med Internet Res.

[60] Kauttonen J, Hannukainen J, Tikka P, Suomala J (2020). Predictive modeling for trustworthiness and other subjective text properties in online nutrition and health communication. PLoS One.

[61] Pedersen EA, Loft LH, Jacobsen SU, Søborg B, Bigaard J (2020). Strategic health communication on social media: insights from a Danish social media campaign to address HPV vaccination hesitancy. Vaccine.

[62] Vollrath K, Thul S, Holcombe J (2018). Meaningful methods for increasing human papillomavirus vaccination rates: an integrative literature review. J Pediatr Health Care.

[63] Skjuve MB, Brandtzæg PB (2018). Chatbots as a new user interface for providing health information to young people. Youth and News in a Digital Media Environment: Nordic-Baltic Perspectives.

[64] Stephens TN, Joerin A, Rauws M, Werk LN (2019). Feasibility of pediatric obesity and prediabetes treatment support through Tess, the AI behavioral coaching chatbot. Transl Behav Med.

[65] Stiefel S (2018). 'the chatbot will see you now': mental health confidentiality concerns in software therapy. SSRN J.

