# Supplementary material for: eHealth Communication Intervention to Promote Human Papillomavirus Vaccination Among Middle-School Girls: Development and Usability Study
Source: JMIR Form Res. 2024 Oct 28;8:e59087. doi: 10.2196/59087 (PMC11555454; doi:10.2196/59087)
Supplement: Multimedia Appendix 1 [file formative_v8i1e59087_app1.docx]

**Table S1 Sample search strategy**

| PUBMED | ((("health communication"[Title/Abstract] AND "hpv vaccin*"[Title/Abstract]) OR "Human papillomavirus"[Title/Abstract]) AND (("program"[Title/Abstract] OR "intervention"[Title/Abstract]) OR "campaign"[Title/Abstract])) AND (("adolescent"[Title/Abstract] OR "youth"[Title/Abstract]) OR "teen*"[Title/Abstract]) | 238 |
| --- | --- | --- |
| CINHAL | AB "health communication" OR AB "health message" AND AB "HPV vaccin*" OR AB "Human papillomavirus" AND AB ( "program" OR "intervention" OR "campaign" ) AND AB ( "adolescent" OR "youth" OR "teen*" ) | 310 |
| Cochrane | ("health communication"):ti,ab,kw OR ("health message"):ti,ab,kw AND ("HPV vaccin*"):ti,ab,kw OR ("Human papillomavirus"):ti,ab,kw AND ("program" OR "intervention" OR "campaign"):ti,ab,kw | 951 |
| PsycINFO | ab("health communication") OR ab("health message") AND ab("HPV vaccin*") OR ab("Human papillomavirus") AND ab("program" OR "intervention" OR "campaign") AND ab("adolescent" OR "youth" OR "teen*") | 113 |
