# Supplementary material for: eHealth Communication Intervention to Promote Human Papillomavirus Vaccination Among Middle-School Girls: Development and Usability Study
Source: JMIR Form Res. 2024 Oct 28;8:e59087. doi: 10.2196/59087 (PMC11555454; doi:10.2196/59087)
Supplement: Multimedia Appendix 2 [file formative_v8i1e59087_app2.docx]

**Table S2 Generic menu of JUMP chatbot**

| **Generic Menu** |  | **1^st^ Block** | |  | | **2^nd^ Block** | | | | | | | | |  | | | **3^rd^ Block** | | | | | | | | | | | | | | | |  | | | **4^th^ Block** | | | | | | | | | | | | |  |  |  |  |  |  |  |  |  |  |
| --- | --- | --- | --- | --- | --- | --- | --- | --- | --- | --- | --- | --- | --- | --- | --- | --- | --- | --- | --- | --- | --- | --- | --- | --- | --- | --- | --- | --- | --- | --- | --- | --- | --- | --- | --- | --- | --- | --- | --- | --- | --- | --- | --- | --- | --- | --- | --- | --- | --- | --- | --- | --- | --- | --- | --- | --- | --- | --- | --- |
|  |  |  | |  | |  | | | | | | | | |  | | |  | | | | | | | | | | | | | | | |  | | |  | | | | | | | | | | | | |  |  |  |  |  |  |  |  |  |  |
| Information on cervical cancer | ⇛ | Definition | |  | |  | | | | | | | | |  | | |  | | | | | | | | | | | | | | | |  | | |  | | | | | | | | | | | | |  |  |  |  |  |  |  |  |  |  |
|  |  | Stage | |  | |  | | | | | | | | |  | | |  | | | | | | | | | | | | | | | |  | | |  | | | | | | | | | | | | |  |  |  |  |  |  |  |  |  |  |
|  |  | Cause | |  | |  | | | | | | | | |  | | |  | | | | | | | | | | | | | | | |  | | |  | | | | | | | | | | | | | |  |  |  |  |  |  |  |  |  |
|  |  | Symptom | |  | |  | | | | | | | | |  | | |  | | | | | | | | | | | | | | | |  | | |  | | | | | | | | | | | | | |  |  |  |  |  |  |  |  |  |
|  |  | Status | | ⇛ | | Interlinked with articles on the status of cervical cancer | | | | | | | | |  | | |  | | |  | | | | | | |  | | | | | | | | | | |  |  |  |  |  |  |  |  |  |  |  |  |  |  |  |  |  |  |  |  |  |
|  |  | Prevention | | ⇛ | | ‘Tears of a bride-to-be’  Card News of the Ministry of Health and Welfare.gif | | | | | | | | |  | | |  | | | | | | | | | | | | | | |  |  |  |  |  |  |  |  |  |  |  |  |  |  |  |  |  |  |  |  |  |  |  |  |  |  |  |
|  |  |  | |  | | Free screening on cervical cancer | | | | | | | | |  | | |  | | | | | | | | | | | | | | | | | | | |  | | | | | | |  | | | | | | |  |  |  |  |  |  |  |  |
| Information on HPV | ⇛ | Definition | |  | |  | | | | | | | | |  | | | | | | |  | | | | | | | | | | | | | | | | | |  | | | | | | |  | | |  |  |  |  |  |  |  |  |  |  |
|  |  | Type | |  | |  | | | | | | | | |  | | | | | | |  | | | | | | | | | | | | | | | | | |  | | | | | | |  | | |  |  |  |  |  |  |  |  |  |  |
|  |  | Cause of infection | | ⇛ | | Does a condom prevent infection? | | | | | | | | |  | | |  | | | | | | | | | | | | | | | | | | | |  | | | | | | |  | | | | | | |  |  |  |  |  |  |  |  |
|  |  |  | | ⇛ | | Do men get vaccinated? | | | | | | | | | ⇛ | | | Watching a drama video | | | | | | | | | | | | | | | |  | | |  | | | | | | | | | | | | | | | |  |  |  |  |  |  |  |
|  |  | Symptom | |  | |  | | | | | | | | |  | | |  | | | | | | | | | | | | | | | | | | | |  | | | | | | |  | | | | | | |  |  |  |  |  |  |  |  |
|  |  | Infection status | | ⇛ | | Checking the risk of infection | | | | | | | | |  | | |  | | | | | | | | | | | | | | | | | | | |  | | | | | | |  | | | | | | |  |  |  |  |  |  |  |  |
|  |  |  | |  | |  | | | | | | | | |  | | |  | | | | | | | | | | | | | | | | | | | |  | | | | | | |  | | | | | | |  |  |  |  |  |  |  |  |
| Information on HPV vaccination | ⇛ | Guide to national free vaccination support project | |  | |  | | | |  | | | | | | | | | | | | | | |  | | | | |  | | | | | | | | | | | |  |  |  |  |  |  |  |  |  |  |  |  |  |  |  |  |  |  |
|  |  | Vaccination target | |  | |  | | | | | | | | |  | | | | | | |  | | | | | | | | | | | | | | | | | |  | | | | | | |  | | | | | | | |  |  |  |  |  |
|  |  | Vaccination schedule | | ⇛ | | My Talk assistant, Jordy | | | | | | | | |  | | |  | | | | | | | | | | | | | | | | | | | |  | | | | | | |  | | | | | | |  |  |  |  |  |  |  |  |
|  |  | Types of vaccine | | ⇛ | | Detailed vaccine types | | | | | | | | | ⇛ | | | Questions about HPV vaccinations | | | | | | | | | | | | | | | |  | | |  | | | | | | | | | | | | | | | |  |  |  |  |  |  |  |
|  |  | Vaccination price | |  | |  | |  | | |  | | | | | | | | | | | | | | | |  |  |  |  |  |  |  |  |  |  |  |  |  |  |  |  |  |  |  |  |  |  |  |  |  |  |  |  |  |  |  |  |  |
|  |  | Effect of vaccination | |  | |  | | | | | | | | |  | | |  | | | | | | | | | | | | | | | |  | | |  | | | | | | | | | | | | | | | |  |  |  |  |  |  |  |
|  |  | Vaccine safety | | ⇛ | | Detailed description of vaccine safety | | | | | | | | | ⇛ | | | vaccination with parents | | | | | | | | | | | | | | | | ⇛ | | | Interlinked with the website ‘Love Plan’ | | | | | | | | | | | | | | | |  |  |  |  |  |  |  |
|  |  | Vaccine adverse reaction | | ⇛ | | Explanation of vaccination adverse reactions | | | | | | | | | ⇛ | | | Call centers for Disease Control and Prevention | | | | | | | | | | | | | | | |  | | |  | | | | | | | | | | | | | | | |  |  |  |  |  |  |  |
|  |  | Guide to vaccination | |  | |  | | | | | | | | | ⇛ | | | Vaccination Helper Website | | | | | | | | | | | | | | | |  | | |  | | | | | | | | | | | | | | | |  |  |  |  |  |  |  |
|  |  | Overseas vaccination status | |  | |  | | | |  | | |  | | | | | | | | | | |  | | | | | | | | | | | | |  |  |  |  |  |  |  |  |  |  |  |  |  |  |  |  |  |  |  |  |  |  |  |
|  |  | Location of vaccination | | ⇛ | | Vaccination institutions in my town | | | | | | | | |  | | |  | | | | | | | | | | | | | | | |  | | | ` | | | | | | | | | | | | | | | |  |  |  |  |  |  |  |
|  |  | Precaution for vaccination | |  | |  | | | |  | | |  | | | | | | | | | | |  | | | | | | | | | | | | |  |  |  |  |  |  |  |  |  |  |  |  |  |  |  |  |  |  |  |  |  |  |  |
|  |  |  | |  | |  | | | | | | | | | |  | | | |  | | | | | | | | | | | | | | |  |  |  |  |  |  |  |  |  |  |  |  |  |  |  |  |  |  |  |  |  |  |  |  |  |
| Vaccination institutions in my town | ⇛ | Guide to vaccination institutions at vaccination helper website | |  | |  | | | | | | | | | |  | | | |  | | | | | | | | | | | | | | |  |  |  |  |  |  |  |  |  |  |  |  |  |  |  |  |  |  |  |  |  |  |  |  |  |
|  |  |  | |  | | | | |  | | | | | | | | | |  | | | | | | |  | | | | | | | | | | |  | | | | | | | | |  | | | | | | | | | | | |  |  |
| Animation to cheer for vaccination | ⇛ | Video to cheer for vaccination | |  | | | | | | | | | | | | | | |  | | | | | | |  | | | | | | | | | | | | | | | | | |  | | | | | | | | | |  | | | | | |
|  |  | Messages for health responsibility | | ⇛ | | If anyone has a family history, provide related articles in advance. | | | | | | | | |  | | | | | | | | | | | | | | | | | | | | | | | | |  | | | | | | |  | | | | | | | |  |  |  |  |  |
|  |  | sex-related conversations with your children | | ⇛ | | Sex education and counseling YouTube | | | | | | | | |  | | | | | | |  | | | | | | | | | |  | | | | | | | |  | | | | | | |  | | | | | | | |  |  |  |  |  |
|  |  |  | | ⇛ | | Interlinked with the website ‘Love Plan’ | | | | | | | | |  | | | | | | |  | | | | | | | | | | | | | | | | | |  | | | | | | |  | | | | | | | |  |  |  |  |  |
|  |  | |  | | |  | | | | | |  | |  | | | | | | | | | | | | | | |  | |  | | | | | | | | | | | | | | | | | |  |  |  |  |  |  |  |  |  |  |  |
| Questions about vaccination | ⇛ | | Key questions | | | ⇛ | | Cross vaccination | | | | | | | | | |  | | | | |  | | | | | | | | | | | | | | | | | |  | | | | | | |  | | | | | | | |  |  |  |  |
|  |  |  |  | | | ⇛ | | Simultaneous vaccination | | | | | | | | | |  | | | | |  | | | | | | | | | | | | | | | | | |  | | | | | | |  | | | | | | | |  |  |  |  |
|  |  |  |  | | | ⇛ | | Vaccination delay | | | | | | | | | |  | | | | |  | | | | | | | | | | | | | | | | | |  | | | | | | |  | | | | | | | |  |  |  |  |
|  |  |  |  | | | ⇛ | | Pretest | | | | | | | | | |  | | | | |  | | | | | | | | | | | | |  | | | | | | |  | | | | | | | | | | | | |  |  |  |  |
|  |  |  |  | | | ⇛ | | Cervical cancer examination | | | | | | | | | | ⇛ | | | | | Explanation of cervical cancer screening | | | | | | | | | | | | | |  | | | | | |  | | | | | | | | | | | | | |  |  |  |
|  |  |  |  | | | ⇛ | | Injection pain | | | | | | | | | | ⇛ | | | | | Adverse reaction after vaccination | | | | | | | | | | | | | |  | | | | | |  | | | | | | | | | | | | | |  |  |  |
|  |  |  | Report side effects | | | ⇛ | | Adverse reaction after vaccination | | | | | | | | | |  | | | | |  | | | | | | | | | | | | | |  | | | | | |  | | | | | | | | | | | | | |  |  |  |
|  |  | |  | | | ⇛ | | Call to report side effect report | | | | | | | | | |  | | | | |  | | | | | | | | | | | | | |  | | | | | |  | | | | | | | | | | | | | |  |  |  |
|  |  | |  | | | ⇛ | | Vaccination safety | | | | | | | | | |  | | | | |  | | | | | | | | | | | | | |  | | | | | |  | | | | | | | | | | | | | |  |  |  |
|  |  | |  | | |  | |  | | | | | | | | | |  | | | | |  | | | | | | | | | | | | | |  | | | | | |  | | | | | | | | | | | | | |  |  |  |
| Difficult terms | ⇛ | | Difficult terms 1 | | |  | |  | | | | | | | | | |  | | | | |  | | | | | | | | | | | | | |  | | | | | |  | | | | | | | | | | | | | |  |  |  |
|  |  | | Difficult terms 2 | |  | |  | | | | | | | | | |  | | | | |  | | | | | | | | | | | | | | | | | |  | | | | | | |  | | | | | | | | | | | |  |
